# An Effective Strategy and Mathematical Model to Predict the Sustainable Evolution of the Impact of the Pandemic Lockdown

**DOI:** 10.3390/healthcare10050759

**Published:** 2022-04-19

**Authors:** Priyanka Harjule, Ramesh Chandra Poonia, Basant Agrawal, Abdul Khader Jilani Saudagar, Abdullah Altameem, Mohammed Alkhathami, Muhammad Badruddin Khan, Mozaherul Hoque Abul Hasanat, Khalid Mahmood Malik

**Affiliations:** 1 Department of Mathematics, Malaviya National Institute of Technology (MNIT), Jaipur 302017, India; priyanka.maths@mnit.ac.in; 2 Department of Computer Science, CHRIST (Deemed to be University), Bangalore 560029, India; rameshcpoonia@gmail.com; 3 Department of Computer Science Engineering, Indian Institute of Information Technology Kota, MNIT Campus, Jaipur 302017, India; basant.cse@iiitkota.ac.in; 4 Information Systems Department, College of Computer and Information Sciences, Imam Mohammad Ibn Saud Islamic University (IMSIU), Riyadh 11432, Saudi Arabia; altameem@imamu.edu.sa (A.A.); maalkhathami@imamu.edu.sa (M.A.); mbkhan@imamu.edu.sa (M.B.K.); mhhasanat@imamu.edu.sa (M.H.A.H.); 5 Department of Computer Science and Engineering, Oakland University, Rochester, MI 48309, USA; mahmood@oakland.edu

**Keywords:** COVID-19, epidemiology, mathematical model, SEIHRD, ordinary differential equations

## Abstract

There have been considerable losses in terms of human and economic resources due to the current coronavirus pandemic. This work, which contributes to the prevention and control of COVID-19, proposes a novel modified epidemiological model that predicts the epidemic’s evolution over time in India. A mathematical model was proposed to analyze the spread of COVID-19 in India during the lockdowns implemented by the government of India during the first and second waves. What makes this study unique, however, is that it develops a conceptual model with time-dependent characteristics, which is peculiar to India’s diverse and homogeneous societies. The results demonstrate that governmental control policies and suitable public perception of risk in terms of social distancing and public health safety measures are required to control the spread of COVID-19 in India. The results also show that India’s two strict consecutive lockdowns (21 days and 19 days, respectively) successfully helped delay the spread of the disease, buying time to pump up healthcare capacities and management skills during the first wave of COVID-19 in India. In addition, the second wave’s severe lockdown put a lot of pressure on the sustainability of many Indian cities. Therefore, the data show that timely implementation of government control laws combined with a high risk perception among the Indian population will help to ensure sustainability. The proposed model is an effective strategy for constructing healthy cities and sustainable societies in India, which will help prevent such a crisis in the future.

## 1. Introduction

Pandemics have plagued humankind for generations. The aftermath of these diseases has a massive impact on the world economies, and the strengths and morality of heavily impacted nations are compromised [1]. India’s rate of development of the COVID-19 pandemic was so rapid that neither the government nor the people had a chance to respond in a sustainable manner. To combat the impact of the pandemic, the administration has had to implement timely and soft policies. 

Health-related concerns have plagued humanity from its inception. The first known pandemic was the Black Death, which appeared in the early 1300s [2]. It was one of the worst pandemics faced by humankind. Older adults and those who have been subjected to psychological stress are most likely to develop this disease [3,4]. Smallpox, which first arose in the early 1500s, had a 50% death rate [5]. Cholera, one of the world’s worst diseases, was the next pandemic to hit the world’s population. It killed more than 1.5 million people. Following this, in 1957, the Asian flu influenza was observed. It killed almost 0.7–1.5 million people [6]. Before that, in 1918, humankind faced another devastating pandemics: the Spanish flu influenza, in which 20 million to 110 million deaths were reported [7]. 

The human immunodeficiency virus infection and the acquired immune deficiency syndrome (HIV/AIDS), which infected more than 70 million people in 1981, has also been reported [8]. In 2003, the world encountered a new series of severe acute respiratory syndrome (SARS) pandemics. Thirty-seven nations and four continents were affected [9,10]. In 2009, the world witnessed a swine flu pandemic, which took 1.56–5.75 hundred thousand lives [11,12]. The SARS pandemic was followed by the three recent pandemics: the Middle East respiratory syndrome coronavirus (MERS), Ebola, and Zika pandemics in 2012, 2013, and 2015, respectively. Two later pandemics took the lives in thousands [13]. Currently, the whole world is witnessing the SARS-CoV-2 (COVID-19) pandemic. More than 212 countries and territories had reported a total of 4.18 million confirmed cases of COVID-19 by 11 May 2020 [14]. Such pandemics have severe, unpredictable, long-lasting, and incalculable effects on many communities around the world.

Consequently, research and contributions to the literature on these factors are essential to achieving the sustainable development goals. An outbreak of the novel COVID-19 disease was reported in Wuhan, Hubei province, on 31 December 2019. The end of February 2020 saw a sudden rise in these instances around the globe. In India, the first case of COVID-19 was reported towards the end of January 2020 [15]. This count has increased with each passing day. Both the first and the second COVID-19 waves have already resulted in significant economic and human losses in developing countries such as India [16]. For example, Delhi, the capital city of India, faced huge losses in tax collection in April compared to the previous year. The government generated INR three thousand million revenues compared to the previous year’s INR 35,000 million [17].

The rest of paper is organized as follows. First, Section 2 provides a recent survey on the mathematical modeling techniques used for COVID-19, followed by the motivations of this research. In Section 3, the conceptualization of the proposed mathematical model SEIHRD is presented. It provides a detailed methodology used to generate the results of this paper. Section 4 concludes with a sensitivity analysis of the time-dependent transmission rate. Section 5 presents the various influences and the associated analysis from the proposed mathematical model, SEIHRD. Lastly, Section 6 presents the conclusions and future scope related to the work of this study.

## 2. Related Work

To anticipate the spread of disease and the number of people who died during a given pandemic period, mathematical modeling and stochastic theory have been used in the past [18,19,20]. In [21], the authors report differences in severity between the first and second waves in Spain.

It has been observed in the literature that mathematical modeling was very efficient in providing better predictions in the past. The same conventional approaches for predicting the spread rate and mortality count have been published in several papers about COVID-19 [22,23,24,25,26,27,28,29].

Sameni [30] has used the classical Susceptible–Infected–Recovered (SIR) model to detect the virus’s spread. Researchers found that quarantine facilities, which contain infected patients, have a favorable impact on the death rate and transmission of the disease. Chatterjee et al. [31] developed the compartmental Susceptible–Exposed–Infected–Recovered (SEIR) model using Monte Carlo simulation. This study also considers hospitalization, ICU requirements, and deaths modeled on SimVoi software. The authors suggested that the immediate implementation of complete lockdown, social distancing, and quarantine can help slow down the pandemic’s spread. Dowd et al. [32] have presented mathematical modeling to predict the death count by considering age and gender as parameters. It has been seen that primarily older adults are affected. Therefore, the age factor of a country can play a vital role in this threat. Countries with a significant population of people aged 65 years or older are at a higher risk. He et al. [33] proposed a mathematical model considering the impact of the pre-symptomatic transmission on the deathrate. It has been observed that the transmission rate can be seen even before the first symptoms become physically visible. Therefore, governments should take appropriate care of pre-symptomatic patients while enforcing measures to control the spread. Banerjee et al. [34] presented the impact of underlying conditions such as heart disease and diabetes on the death rate. Vasily Zhang et al. [35] proposed a segmented Poisson model to analyze the available daily new cases in six Western countries.

Pandey and colleagues [36] used the Susceptible–Exposed–Infected–Recovered (SEIR) and Regression models to predict the spread for governments and doctors in preparing their plans. Mohamadou et al. [37] published a reviewed paper covering various papers on mathematical modeling, artificial intelligence, etc., for predicting and managing COVID-19. The development of mathematical models and theories to predict the spread of COVID-19 is an essential part of a sustainable response to the pandemic. It also aids in policy formulation for future COVID-19 waves by analyzing the present responses of developing countries such as India. A deterministic compartmental model is also considered in this study. These models are aligned with the mathematical modeling literature. The classic deterministic model available in the literature is the Susceptible–Infected–Recovered (SIR) model. However, the most frequently employed deterministic mathematical model for COVID-19 was the Susceptible–Exposed–Infected–Recovered (SEIR) model. The SEIR model was employed in different ways. Some researchers used it in combination with other statistical models. At the same time, some authors modified it to fit the new COVID-19 rules for infections specific to different geographic conditions and the related available data available [22,38,39]. The popularity of this model may be attributed to its adaptable nature. It is easy to add more parameters and states to these kinds of models as compared to statistical and machine-learning-based models. To characterize the multi-dimensional COVID-19 issue more precisely, researchers may prefer compartmental models. An SEIR model was implemented to investigate the dynamics of the spread and prevention of COVID-19. Uncertainty in case observation was also considered in this model by overtly modeling a Poisson observed process of new symptomatic COVID-19 cases. Using Monte Carlo simulation, a geometric random walk method was employed to model the transmission and simulated the transmission rate over time. Yang and colleagues [40] incorporated China’s migration population data and COVID-19 data into the SEIR model to predict that the pandemic peaked in late February and declined in last April in China. They also employed artificial intelligence techniques used on 2003 SARS statistics to verify their outcomes. A modified SEIR model was proposed by Li et al. [41]. They considered a six-state mathematical model, namely the Susceptible–Exposed–Infected–Quarantined–Potential Victims–Recovered (SEIQDR) model. Mathematical modeling has been used to forecast and make decisions regarding this disease in a number of articles [42,43,44]. In a similar vein, many review papers are noted for providing comprehensive information [45,46,47,48]. Finally, this comprehensive review of current literature revealed the variety of work that has been accomplished in forecasting and regulating the spread of the disease. 

### 2.1. Objectives

COVID-19’s mutation and the paucity of immunization may cause the illness to spread rapidly in underdeveloped countries such as India, resulting in significant economic and human losses. Therefore, a prediction of the present state of the COVID-19 epidemic spread in India can help to understand how different situations may affect the potential outcome of the epidemic. The government can accordingly plan for the mitigation of the spread and implement policies for the betterment of its citizens and the economy, anticipate hospital needs, and devise testing strategies. The proposed mathematical model (SEIHRD) and the related analysis presented in this paper are modified as per a real scenario specific to different states of India, which is different from the existing models in the literature in multiple ways. It is considered essential to develop models based on various parameters to analyze the pandemic situation in the world. At the same time, due to vast geographical and demographical differences, it is essential to carry out the analysis specific to a region/country.

### 2.2. Contributions

Using a time-dependent modification of the conventional SEIR model, this study analyzes and models the dynamics of the unique coronavirus pandemic and accompanying lockdowns that are specific to Indian society. The proposed SEIHRD model consists of six states and determines the effect of preventive measures through transmission probability and key epidemic parameters, viz., the latent time, hospitalization rate, time-dependent mortality rate, and time-dependent recovery rate, in a relatively reliable way for India. Table 1 lists out the different points of the classical SEIR model and the proposed model. The highlights of the model are as follows:

- Unlike the classical Susceptible–Exposed–Infected–Removed (SEIR) model, the proposed SEIHRD model has six states that differentiate between diagnosed and non-diagnosed individuals.
- In this study, the authors consider two different transmission rates for a susceptible person to be infected and a susceptible person to become a carrier, respectively. This discrimination is significant because the diagnosed individual is isolated and less likely to spread the highly infectious coronavirus disease. In the absence of strong visible symptoms, it becomes difficult to test an individual for COVID-19 unless that individual has come in contact with a positive confirmed case.
- The mortality rate and recovery rate are taken as time functions as the disease progresses in India. This choice is data-driven. A change in mortality rate and recovery rate has been observed since the first case in January 2020.
- In addition, a time-dependent transmission rate, which incorporated government control policy and public perception of risk, has been investigated along with the conceptual model to perform a sensitivity analysis in Indian context.

**Table 1:** Difference between the classical and the proposed model.

Sr. No.	Classical Model	Proposed Model
1	Classical SEIR model has four states	The proposed model has six states
2	The classical model SEIR do not differentiate be-tween diagnosed and non-diagnosed individual	The proposed model discriminates between diagnosed and non-diagnosed individual
3	Pre-symptomatic transmission is not taken into account	Pre-symptomatic transmission is taken into account
4	SEIR is not dependent on time	Various transmission rates are time-dependent
5	Governmental control and public perception of risk of the disease not included	Governmental control and public perception of risk of the disease are taken into account

The proposed model takes into account six states: Susceptible, Exposed and Undetected, Infected and Detected, Hospitalized, Recovered, and Dead. The proposed model is different from existing models for the following reasons: (i) it discriminates between infected individuals who are diagnosed and those who are not diagnosed; (ii) it employs two different transmission rates for diagnosed and non-diagnosed individuals; (iii) the proposed model considers the time dependency of the mortality rate and the recovery rate depending on India’s poor and congested societies. This study also implements a time-dependent transmission rate to investigate the impact of governmental control policy action and people’s perception of risk reaction to predict the course of the evolution of COVID-19 in India.

## 3. Mathematical Modeling of COVID-19

The use of dynamical equations in mathematical modeling, as opposed to statistical approaches, can often reveal vital information about epidemic dynamics. When basic epidemiological parameters are unknown or possibly uncertain, a more systematic approach is required to describe the Susceptible–Infected–Recovered (SIR) model, a classical compartmental model in epidemiology, which represents the spread of an infectious disease through a population, what fraction of the population may become infected and when. This model was developed in the twentieth century in 1927 as part of [49]’s exploration of epidemiology theory.

### 3.1. Proposed Model: SEIHRD

The Susceptible–Exposed–Infected–Removed (SEIR) model [48] is an extension of the SIR model. For many infectious diseases such as 2019-nCoV, there is a significant incubation period during which individuals have been infected but are not yet infectious themselves. During this period, the individual is in compartment E (for exposed). In this section, a new mathematical model SEIHRD is proposed, a modified form of the classical SEIR model, to analyze and predict the evolution of COVID-19’s spread in India. The proposed model SEIHRD consists of six states, namely:

- The susceptible compartment contains the population that is susceptible to the highly infectious novel coronavirus.
- The exposed compartment consists of a fraction of susceptible populations comprising infected and asymptomatic infected individuals (carrier/latent class). They are not yet detected but are infectious.
- The infected state comprises the fraction of exposed people who are asymptomatic and symptomatic infected individuals. However, they are detected and infectious.
- The hospitalization compartment consists of a fraction of the infected population that is hospitalized.
- The recovered state contains the fraction of hospitalized people who have recovered from the infectious disease.
- The dead state consists of a fraction of hospitalized people who have died from the infectious disease.

The assumptions are based on India’s existing conditions and scenarios known from media and updates provided by the Indian Council of Medical Research (https://www.icmr.gov.in/ accessed on 30 November 2021) on a day-to-day basis during the first and second waves of COVID-19 [50]. The computational procedure is given in the following steps:

Step 1: Downloading the data from www.COVIDindia.org and read them.
Step 2: Initialization of parameters: Recovered daily, Deaths daily, and Confirmed cases daily.
Step 3: Fitting the proposed SEIHRD model to the real data with a function.
Step 4: Estimation of the parameters such as fitted infection rate, transmission rate, recovery rate, rate at which individual is admitted to hospital, etc.
Step 5: Simulate the epidemic outbreak based on the fitted parameters.
Step 6: Comparison of the fitted data and the accurate data.

**Figure 1:**
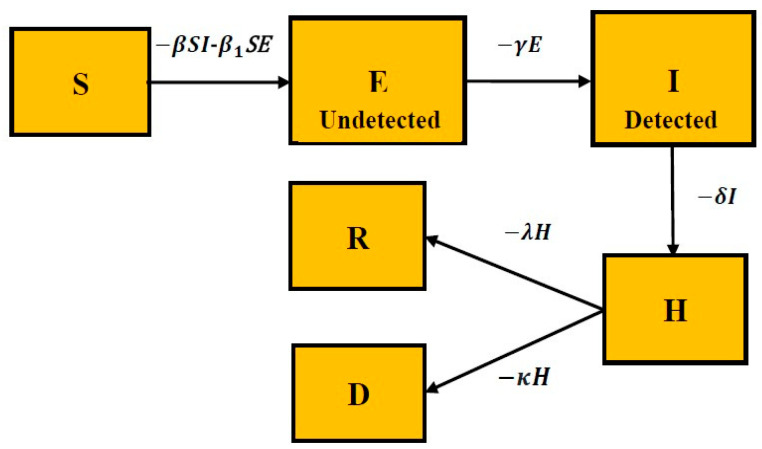
The proposed SEIHRD model.

The discrimination between diagnosed and not-diagnosed individuals is essential in the highly infectious COVID-19 disease because the diagnosed individual is quarantined and is less likely to spread the disease. It also employs two different transmission rates (*β* and *β*_1_, both dependent on governmental policies) for diagnosed and non-diagnosed individuals. The Indian COVID-19 data drive the addition of the new Hospitalized compartment. The number of confirmed active cases in the Indian COVID-19 data are all those that are hospitalized. The relations between all these states are pictorially represented in the form of a diagram as shown in Figure 1. 

Table 2 presents the data regarding the number of active and recovered cases in India and four different states as of 11 May 2020. These data were used to implement the proposed model.

#### 3.1.1. Assumptions

- A number of assumptions have been made based on the availability of data in India and periodic World Health Organization press releases: the natural death rate and birthrate do not change the population structure significantly across all compartments, and they are therefore taken to be constant so that the population size is constant.
- Unlike the usual SEIR model, the Latent population (E) here is asymptomatic but infectious and undetected or not diagnosed.
- The Infectious population (I) is asymptomatic, as well as infectious and detected/diagnosed.
- Upon coming into contact with Infected (I) or Latent (E) individuals, Susceptible (S) individuals become contagious.
- Only Infectious cases (I) fill up the hospitals and can lead to a higher fatality rate due to shortage of available care.
- Individuals are immune after recovery (R).

#### 3.1.2. Mathematical Model

The COVID-19 dynamics relevant to the Indian context can be modeled by the following set of nonlinear ordinary differential equations:

$$\frac{dS\left(t\right)}{dt}=-\beta \frac{S\left(t\right)I\left(t\right)}{N_{pop}}-\beta_{1}\frac{S\left(t\right)E\left(t\right)}{N_{pop}},$$

$$\frac{dE\left(t\right)}{dt}=-\gamma E\left(t\right)+\beta \frac{S\left(t\right)I\left(t\right)}{N_{pop}}+\beta_{1}\frac{S\left(t\right)E\left(t\right)}{N_{pop}},$$

$$\frac{dI\left(t\right)}{dt}=\gamma E\left(t\right)-\delta I\left(t\right),$$

(1) $$\frac{dH\left(t\right)}{dt}=\delta I\left(t\right)-\kappa \left(t\right)H\left(t\right)-\lambda \left(t\right)H\left(t\right),\;$$

$$\frac{dR\left(t\right)}{dt}=\lambda H\left(t\right),$$

$$\frac{dD\left(t\right)}{dt}=\kappa \left(t\right)H\left(t\right).$$

The constant *N_pop_* = *S* + *E* + *I* + *H* + *R* + *D* and represents the total number of populations in a certain region; it is assumed to be constant. The other parameters of system (1) are listed in Table 3.

Transmission risks (*b* and *b*_1_) can be explained with the following example: the first (or primary) case within a defined group (such as a school or family) is identified, and the people infected by this individual (called secondary cases) are documented. If the number of susceptible individuals in the group is n and the number of secondary cases is x, then an estimation of the transmission risk is *b* (or *b*_1_) = x/n. The effective contact rates or transmission probabilities *β* and *β*_1_ are used to model the different actions of control policies for COVID-19’s spread in India. The total number of contacts (*k*) of an infected person in I is assumed to be less than the total number of contacts (*k*_1_) of an exposed person in *E,* assuming that people generally tend to avoid contacts with those showing symptoms and that those individuals have been quarantined. Additionally, these two quantities, *k* and *k*_1_, also depend upon the government control policy measures such as lockdown or social distancing. Before 24 March 2020, the values *k* = 10 and *k*_1_ = 20 are set. After 24 March 2020, *k* = 5 and *k*_1_ = 10 are chosen assuming that people are following the principles of social distancing and personal hygiene measures under a lockdown scenario. Additionally, b is found by fitting the real-time active cases data to an exponential curve in MATLAB. The values for b (before and after 24 March 2020) are given in Table 4. Therefore, *β*_1_ is typically larger than *β*. The parameters’ recovery rate λ(t) and mortality rate *κ(t)* are taken to be dependent on time in this study to analyze the spread of COVID-19 in India. These are modeled by Equation (2):

$$\kappa \left(t\right)=\kappa_{0}\exp\left(-\kappa_{1}t\right),$$

(2) $$\lambda \left(t\right)=\frac{\lambda_{0}}{(1+\exp\left(-\lambda_{1}\left(t-\tau \right)\right)},$$

where *κ*_0_*, κ*_1_*, λ*_0_, and *λ*_1_ are dimensionless constants and *τ* has the dimension of time. These parameters are the fitted coefficients. The idea behind these functions is that the death rate should become close to zero as time increases while the recovery rate converges towards a constant value.

The classical fourth-order Runge–Kutta method solves the nonlinear ordinary differential equations described in (1).

#### 3.1.3. Dataset Description

Time-series datasets are collected for the real-time prediction of COVID-19 cases for India. Daily confirmed cases from 30 January through 11 May 2020 are considered for fitting the model. Public data were obtained from the source https://www.COVID19india.org/ (accessed on 12 May 2020) [50] and correspond to the Confirmed cases, Recovered cases, and Deceased cases. The model was fitted in a nonlinear approach by calculating the normalized least-squares error of the model approximations and the active infected reported cases. The fitted parameters are summarized in Table 4.

#### 3.1.4. Time-Dependent Transmission Rate

**Figure 2:**
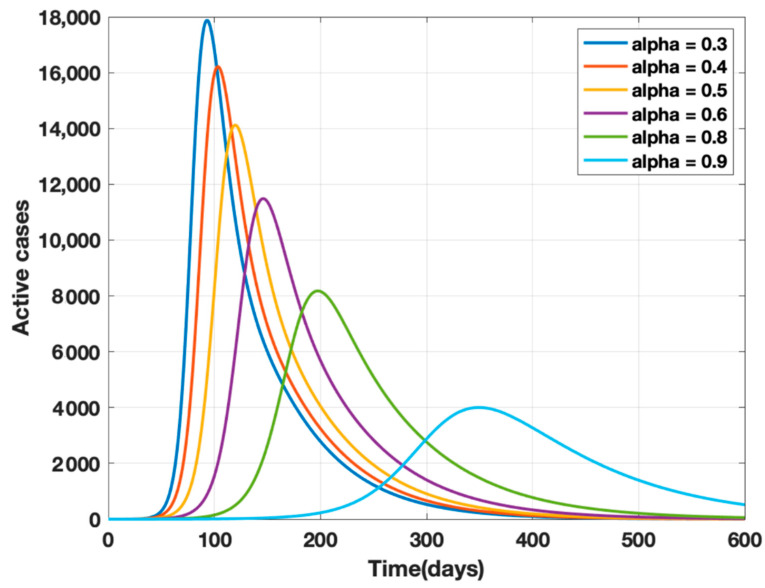
Sensitivity analysis of α and k for α = 0.3, 0.4, 0.5, 0.6, 0.7, 0.8, and k = 10, ρ^−1^ = 10 days.

**Figure 3:**
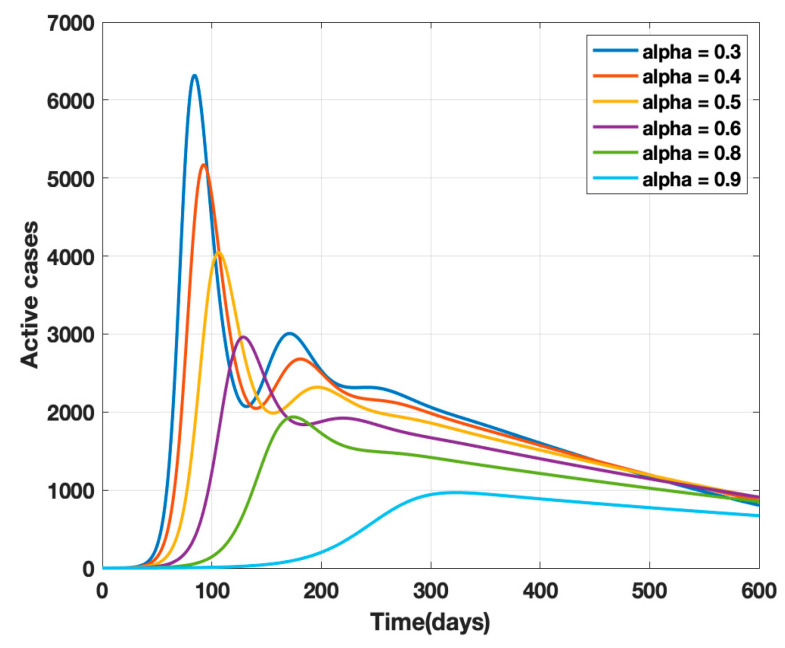
Sensitivity analysis of α and k for α = 0.3, 0.4, 0.5, 0.6, 0.7, 0.8, and k = 100, ρ^−1^ = 10 days.

**Figure 4:**
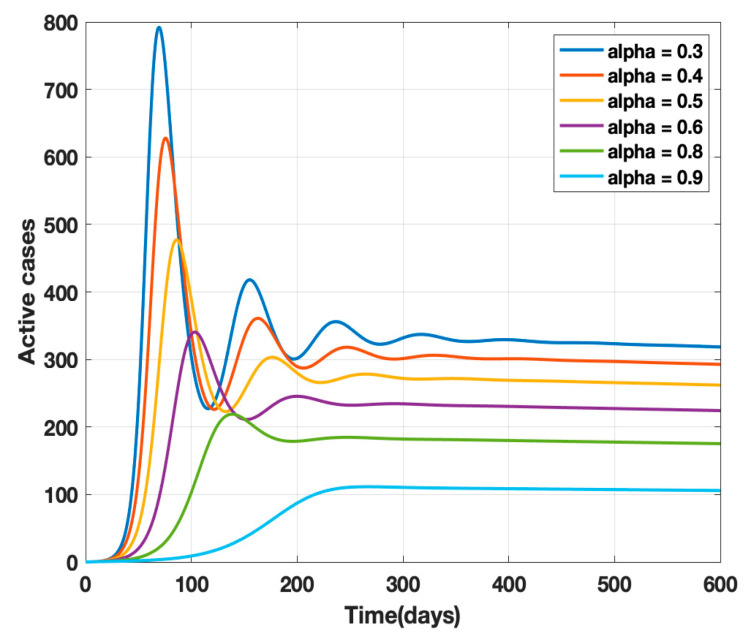
Sensitivity analysis of α and k for α = 0.3, 0.4, 0.5, 0.6, 0.7, 0.8, and k = 100, ρ^−1^ = 10 days 0.5, 0.6, 0.7, 0.8, and k = 1000, ρ^−1^ = 10 days.

In the proposed model described by system (1), two transmission rates (*β* and *β*_1_) are considered. To incorporate the impact of government policies and public actions to control the highly infectious 2019-nCoV disease, the following equation for transmission rates can be integrated with the proposed model, which includes the impact of both governmental control actions and public perception of risk from the disease [51,52].

(3) $$\beta \left(t\right)=\beta_{0}\left(1-\alpha \right)\;{\left(1-\frac{P\left(t\right)}{N_{pop}}\right)}^{k},\;$$

where β_0_ is the initial transmission rate and *P(t)* is the public perception of risk, which increases as the number of cases rise, particularly the number of cases of death, and eventually decreases with time when there are no new cases. When the public perception of risk increases, people are scared and adopt health safety measures, such as frequent hand washing, social distancing, and using face masks, more stringently. *k* is a constant that denotes the strength of public perception of risk. *P(t)* is given as follows:

(4) $$\left(t\right)=\kappa H\left(t\right)-\rho P\left(t\right).$$

where *κ* is the mortality rate and *ρ* − 1 is the mean period of the impact of the number of deaths on the public, i.e., the factor controlling the strength of public perception. The transmission rates *β* and *β*_1_ are replaced by *β(t)* given by Equation (4) in the proposed model (1) and a sensitivity analysis of α (governmental policy action) and k (strength of public action) is carried out as shown in Figure 2 and Figure 3. It is observed from the analysis that to combat the pandemic, both governmental policy actions and public perception of risk are required. Further, the early approach of the peak or delaying the peak depends on the healthcare capacities and management capabilities of a densely populated country such as India. For example, it was found empirically that when both governmental control measures (α = 0.8) and a fair strength of public perception of risk (*k* = 1000) exist, the curve for the daily active cases will flatten out around 200 days from the start of the epidemic, as shown in Figure 4. The main objective behind such an analysis was to contribute to the conceptual understanding of the proposed mathematical model and to demonstrate the extent of impact of government policy measures and public perception of risk on the progress of the highly infectious disease COVID-19.

## 4. Results

The data for cumulative confirmed cases, cumulative recovered cases, and cumulative deceased COVID-19 cases of India were categorized into two temporal categories: (a) 30 January 2020 to 24 March 2020 and (b) 25 March to 4 May 2020. The analysis was carried out on these two categories of data to study the impact of lockdown in India. Further, a prediction for daily confirmed active cases after 4 May 2020 is made based on the fact that government control policies and public health safety measures were in existence, i.e., *k* = 5 and *k*_1_ = 15.

**Figure 5:**
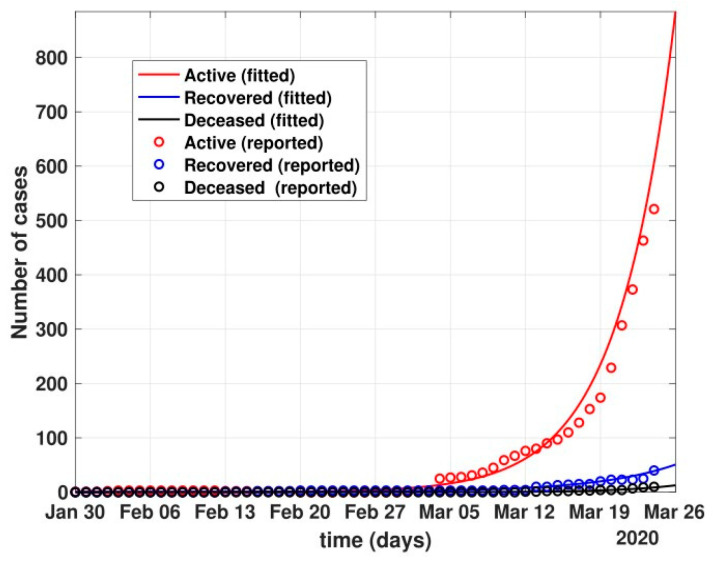
Fit of the proposed model (1) for the number of COVID-19 cases before lockdown, i.e., 24 March 2020.

**Figure 6:**
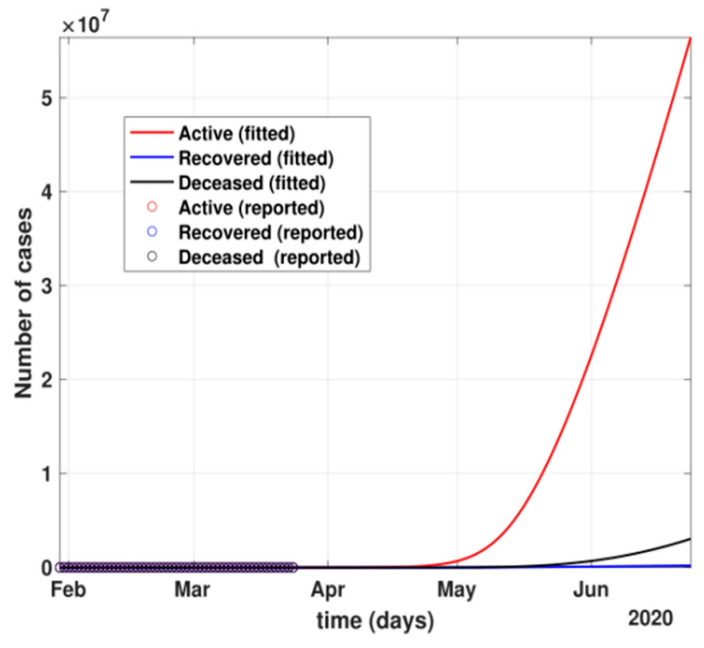
The prediction for active cases after 24 March 2020 with no government control or public health safety measures.

**Figure 7:**
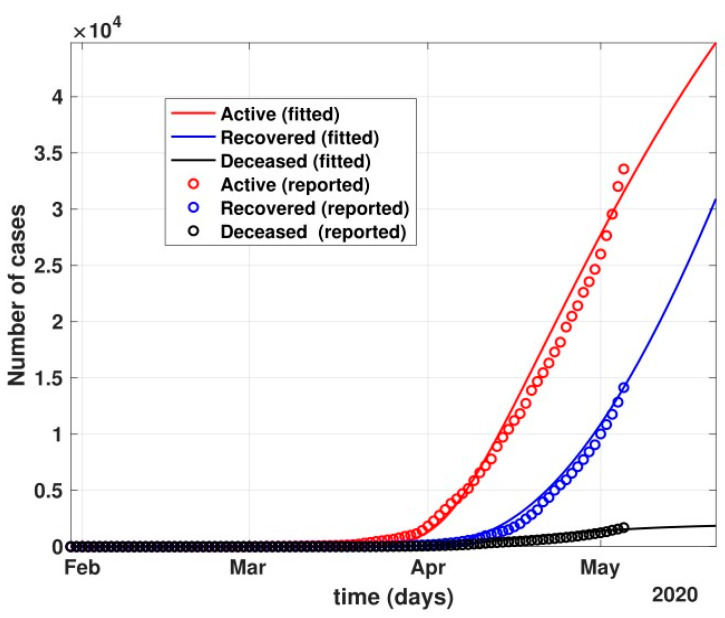
The prediction of fit of the proposed model described by system (1) for COVID-19 data until 4 May 2020.

**Figure 8:**
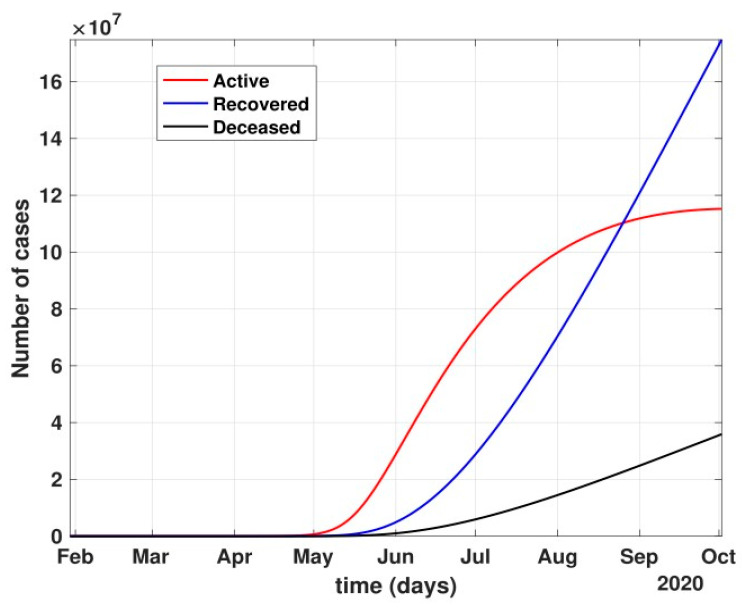
Prediction of the active number of cases after 24 March 2020 with partial governmental control policy and public health safety perception.

**Figure 9:**
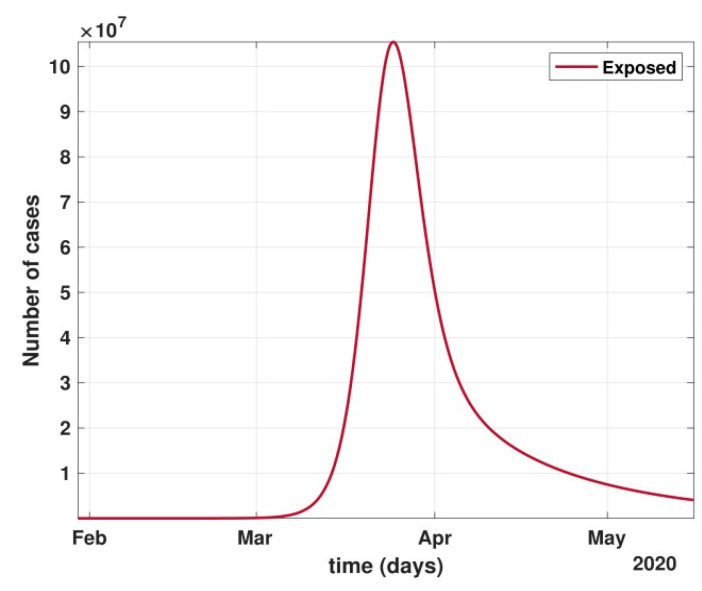
Number of cases in the Latent/Carrier class dropped significantly during April and May, i.e., the period of strict lockdown in India.

**Figure 10:**
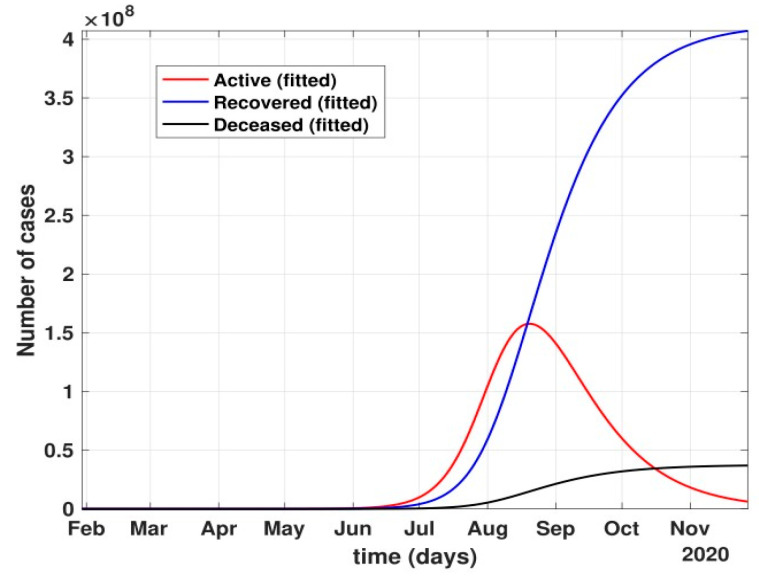
Prediction of the number of active cases after strict lockdown ended and a phased re-opening of the country was in place, i.e., after 4 May 2020.

**Figure 11:**
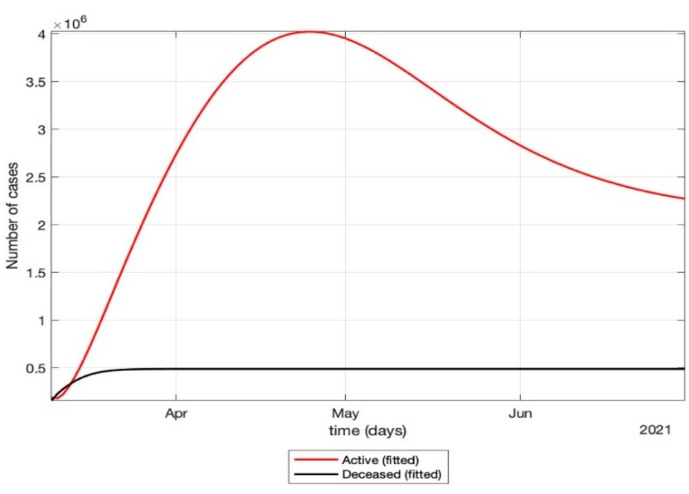
Prediction of the proposed model (1) for the number of COVID-19 cases in 2021 (second wave).

**Figure 12:**
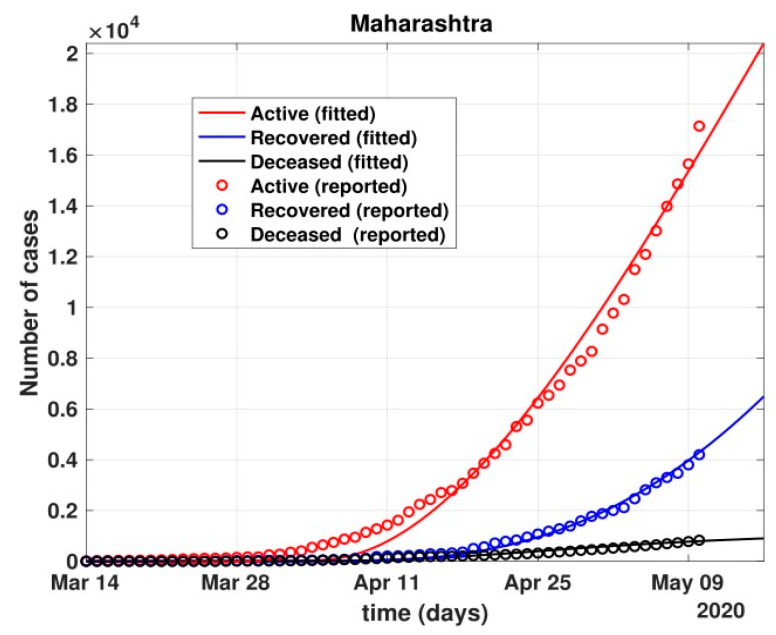
Fit of the proposed model (1) for the number of COVID-19 cases in Maharashtra.

**Figure 13:**
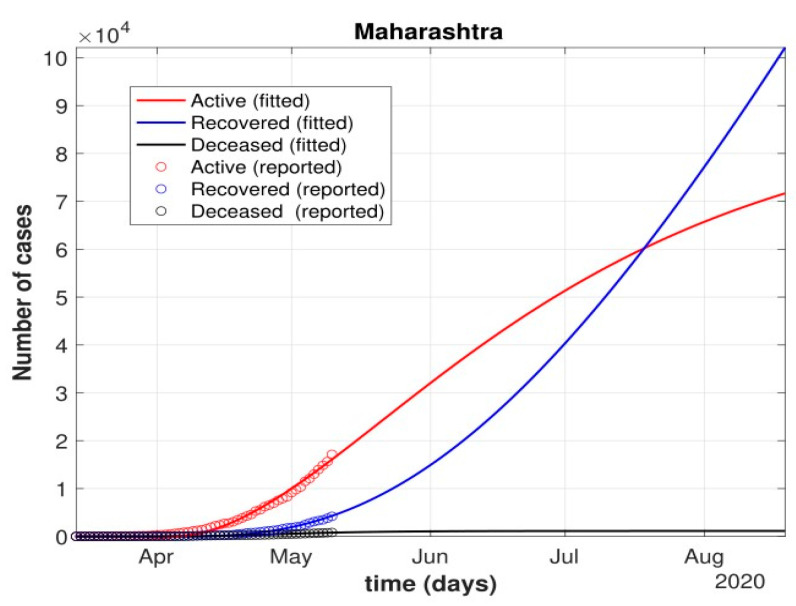
Prediction of the number of cases in Maharashtra using the proposed model.

**Figure 14:**
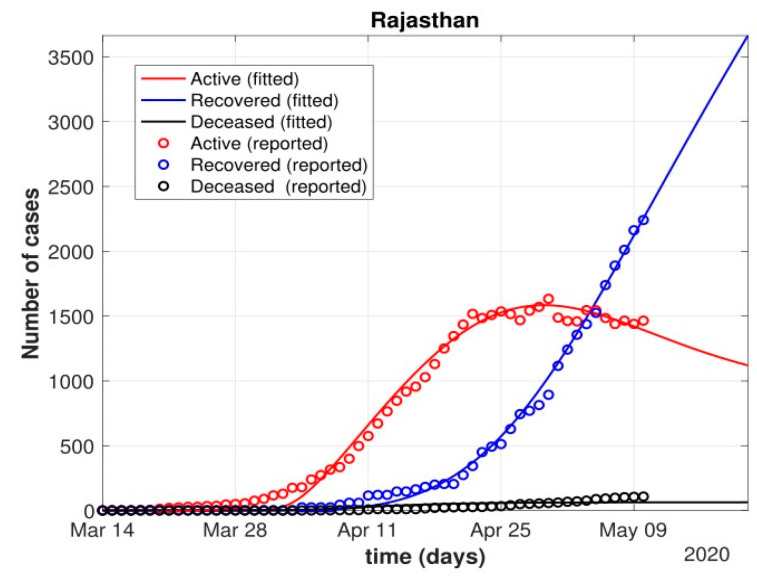
Fit of the proposed model (1) for the number of COVID-19 cases in Rajasthan.

**Figure 15:**
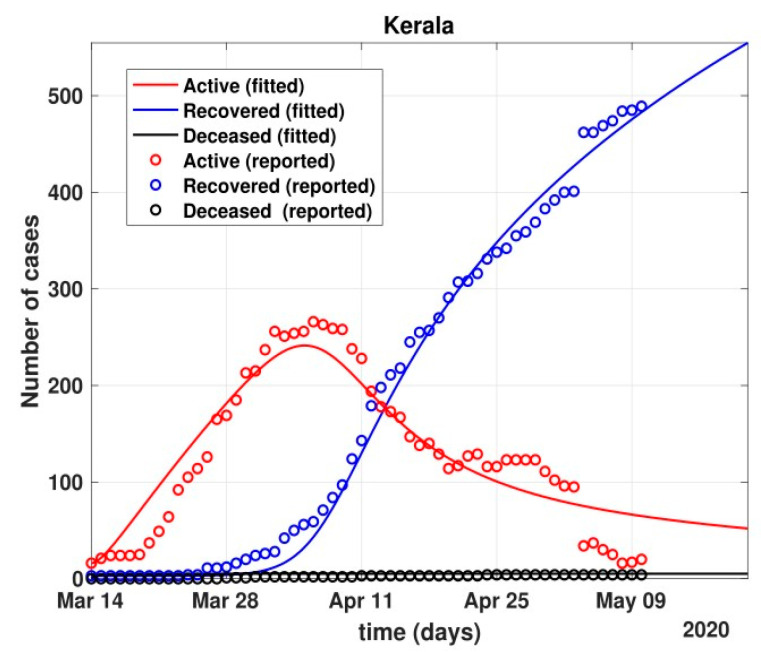
Fit of the proposed model (1) for the number of COVID-19 cases in Kerala.

**Figure 16:**
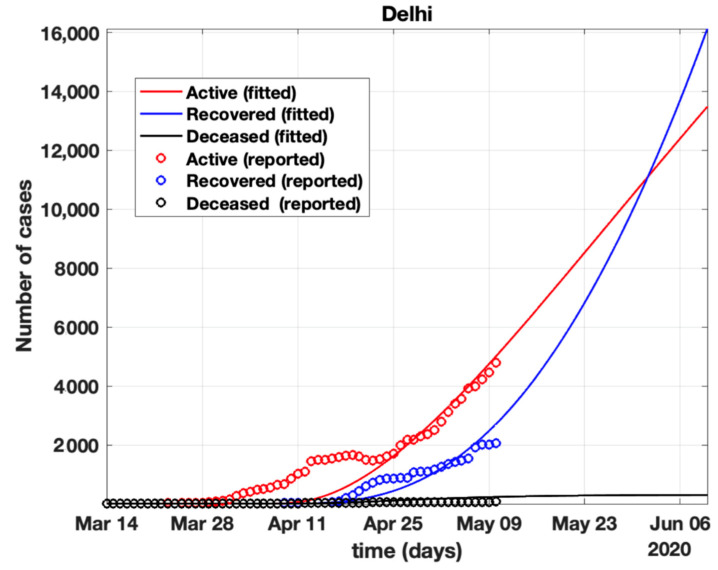
Fit of the proposed model (1) for the number of COVID-19 cases in Delhi.

- The figure also shows the approximate huge number of active cases in the scenario with no government and no public reactions. The proposed model fit for number of cases before 24 March 2020 can be seen in Figure 5. Before 24 March, when the total number of active cases doubled every three days, without any control policy measures and slow but existing (as is visible from the transmission rates *β* and *β*_1_ values) public health perception such as washing hands regularly, etc., the peak of active cases was nowhere to be seen in near four months, as shown in Figure 6.

- Between 24 March and 4 May 2020, a time of two consecutive strict lockdowns in the country, there was a steady growth in the total number of active cases. When these data were fitted to the proposed model described in system (1) (shown in Figure 7), the curve of cumulative active cases intersected the curve of recovered cases in June 2020 and started further flattening, as is visible in Figure 8.

- The total number of active cases again increased in April 2021, as shown in Figure 9. Further, with the governmental control policies and public perception of COVID-19, the appropriate behavior was seen in terms of the number of active cases decreasing in the month of June 2021. As shown in Figure 10, the number of active cases and number of deaths during the second wave were much higher compared to that of the first wave, even with government control policies.
- Further, analysis for four Indian states, namely Maharashtra, Rajasthan, Kerala, and Delhi, was carried out. The proposed model was fitted to the data of COVID-19 confirmed cases, recovered cases, and deceased cases until 11 May 2020, from these four states, as shown in Figure 11, Figure 12, Figure 13, Figure 14, Figure 15 and Figure 16, respectively. Note that after 4 May 2020, the lockdown was opening up in many parts of these four states [12].
- The model when applied to the data of the number of coronavirus cases in 2021 in India. It was found that for the second wave, the transmission rate was quite high for both symptomatic and asymptomatic individuals, i.e., 0.7 and 0.96, respectively. Further, the mean time in which an infected individual entered the hospitalized compartment was found to be three days.

The details of experimental results involve the prediction of the active number of cases before and after strict lockdowns in India due to the COVID-19 pandemic in 2020. The formulation of the mathematical model in the form of ordinary differential equations for the evolution of COVID-19 with respect to the dynamics of India was well fitted with the existing data, as described in Section 3. However, the model gave performance variation for the second wave of COVID-19 in India. This is due to the fact that the parameters such as the amount of medical help available and the amount of oxygen supplies affecting the evolution of the pandemic in India during the second wave were not considered in the mathematical model due to lack of data availability.

## 5. Discussions

- After 24 March, if the country was to have policies of governmental control, including the shutdown of educational institutes public recreational zones such as gyms, spas, and multiplex theatres, and public health safety measures such as compulsory wearing of mask and frequent hand washing then the analysis for such a scenario is carried out by first estimating the value of b = 0.0543 from the number of active cases till 24 March by fitting the real data to an exponential curve in MATLAB and then setting *k* = 5 and *k*_1_ = 15 to obtain the transmission rates from an infected individual to a susceptible individual and from a latent carrier (E) to a susceptible individual. It may be noted that the reason behind the choice of *k*_1_ = 15 is that most of the residential areas in India are densely populated with 7–15 people living in an average of one household. The curve of the active number of cases begins flattening in August with far more active cases, as seen in Figure 9 than the healthcare capacity. The result of such an analysis is far from the reality now. This suggests that the concept of partial control actions from the government among the public are not at all sufficient to control this pandemic in a densely populated and vastly dynamic country such as India. Therefore, a long period of strict lockdown (25 March to 4 May 2020) was essential to keep the highly infectious disease situation under control.
- As seen from the fitting parameters in Table 4, the values of recovery rate and the mortality rate (fitted using Equation (2)) are very close to real rates of 27.52% [12] and 1.89% [12], respectively, from 24 March until 4 May 2020.
- The value of *δ* was found to be 0.13, which implies that only 13% of the total infected people moved from the state *I* to *H*. This implies that only 13% of the infected detected people were taken to the hospital, and rest of them did not require hospitalization, as they were asymptomatic or had mild symptoms. This is in line with the recent report of the Indian Council of Medical Research [12] that 85% of all COVID-19 confirmed cases in India were asymptomatic or had mild symptoms and mostly did not require hospitalization.
- Under policy-controlled measures such as lockdown and public health measures such as social distancing, frequently washing hands and the use of masks all the time will be beneficial in bending the curve of cumulative Active cases sooner, as is visible in Figure 8, under the strict lockdown phase from 25 March to 4 May 2020. It is clearly seen from Figure 8 that under strict policy measures as well as from a public health perspective, the curves of cumulative Active cases intersect the curve of the recovered cases in June and then start further flattening.
- An analysis of the proposed model was carried out under the presence of partial governmental policy measures and effective public health safety measures such as frequent hand washing, the use of face masks all the time, and maintaining social distancing among the general public, after the period of strict lockdown (after 4 May 2020). Value of *b* was obtained and was found to be equal to 0.0645 by fitting with the active cases data till 11 May and thereafter setting *k* = 5 and *k*_1_ = 15. The result, as shown in Figure 10, suggests that it would take a long time to further bend the curve of Active cases in India by August 2020. This delay may be a result of a sudden increase in the number of confirmed cases in India between 5 May and 11 May. This increase may be due to the huge migration of migrant workers from one city to another, in addition to the easing out of the lockdown in many places across the country. The increase in the number of daily confirmed cases between 5 May and 11 May 2020 may also be a result of increased testing of migrant workers. It may be noted that the choice of *k*_1_ = 15 in order to carry out such analysis is due to the fact that most of the residential areas in India are densely populated, with 7–15 people on average living in one household.
- Lockdowns have certainly delayed the peak, or eliminated the peak altogether, in cases of stringent public health measures such as social distancing, washing hands frequently, and the use of masks all the time.
- There are a large number of people in the carrier class/latent population (E), which saw a drastic decline during the strict lockdown period from 24 March to 4 May 2020 (as shown in Figure 10). These individuals are asymptomatic and infectious but the virus is not yet detected in them. This may be attributed to the compactness of the domestic settlements in and around the hotspot virus-affected areas of India. This re-implies that in the absence of a vaccine or an anti-viral drug, both government policy measures and public health safety perspective (controlled by *k* and *k*_1_) should be in place to control spread of the highly infectious COVID-19 in a multi-dynamical country such as India.
- The states of Rajasthan and Kerala are already witnessing the downfall of the coronavirus epidemic, as seen in Figure 14 and Figure 15. On the other hand, the states of Delhi and Maharashtra will take some time before their number of recovered cases is greater than their active cases, as is visible in Figure 13 and Figure 16.
- Finally, a prediction of the number of COVID-19 cases during the second wave in 2021 is depicted in Figure 11. The graph shows a peak in the coronavirus cases at the end of April and a decrease in number of cases in June 2021. This prediction is comparable to the real scenario during the second wave in India. This prediction is based on the government control measures in place, as well as the fact that the public safety perspective is higher. However, the number of active cases, i.e., individuals who require hospitalization, is very high, and the number of deaths is also very high compared to the first wave, even with various government control policies in place.
- The calculation is primarily based on the two different effective contact rates for asymptomatic and symptomatic individuals. These are proportional to the number of contacts that an individual has per day depending on government policies such as lockdown and closure of public places. The other two parameters are time-dependent mortality and recovery rates, which are data-driven and may depend on dynamic factors such as geographical location, weather conditions, and settlements specific to India. The spread can be controlled by controlling the first two parameters by strict government policy and responsible behavior by the public, which could bring down the first two parameters significantly. This is evident in the case of both the first and the second waves of COVID-19.
- It may be noted that the parameters of transmission rates and mean time of hospitalization for an individual, when compared to the first wave of COVID-19 in India, are quite high, which may be the cause of a huge number of active cases during the second wave in India putting a pressure on the medical resources of the country even during the second wave of COVID-19 in India.

## 6. Conclusions

In this study, a new mathematical model, SEIHRD, was proposed to analyze the evolution of COVID-19’s spread in India. This new model appropriately incorporates the inherent influence of infectious latent and infected cases on the entire course of action of the novel coronavirus epidemic, which is difficult for traditional statistical analysis. Based on the public data of COVID-19 cases in India, key parameters such as the transmission rate, latent time, hospitalization rate, mortality rate, and recovery rate are more reliable. In addition, a time-dependent transmission rate was investigated to predict the spread of COVID-19 in India. Results demonstrate the evolution of COVID-19 during lockdowns in India during the first and second waves of COVID-19. Results show that lockdowns in India have successfully helped the country to shift the peak of the epidemic.

Meanwhile, the government utilized the time to create mass awareness about the preventive measures amongst the general public and to pump up the vaccination drive. It was also noted that a good equilibrium between government control policy and strong public perception of risk is essential to further control and coexist with the disease. However, results should be interpreted with care, as projections of the epidemic are highly dependent on the quality of data, producing significant variations in trends with minor changes in observed values. The proposed models have certain limitations, such as the lack of granular data on contact tracing in India and the amount of medical care available, especially during the second wave of COVID-19 in India. The model does not consider humans’ differential susceptibility and infectivity to COVID-19 infection, nor does it consider spatial heterogeneity. This study can be applied to different countries using their respective parameters. The authors wish to enhance the conceptualization of the proposed SEIHRD model in the near future by integrating the factors associated with new variants of the virus, data on the amount of medical care available, and the effect of vaccination in several age groups specific to India. Futures studies could investigate the impact of vaccination on the evolution and spread of the COVID-19 pandemic with respect to India’s geographical and economical dynamics.

## Figures and Tables

**Table 2:** Statistical information of the data.

Place	Total Number of Active Cases	Total Number of Recovered Cases	Total Number of Deaths
India	30,889	12,410	1522
Maharashtra	12,974	2115	548
Rajasthan	1500	2250	40
Kerala	34	462	3
Delhi	4549	1362	64

**Table 3:** The different parameters of the proposed model described by system.

S.no.	Coefficient	Meaning	Remark
1	*β* = *k.b*	Transmission rate from S to I.	Estimated
2	*k*	Total number of contacts, (effective), per day of an infected person.	Policy dependent
3	*b*	Probability of infection between infectious and susceptible individual.	To be fitted
4	*β*_1_ = *k*_1_*.b*_1_	Transmission rate from S to E.	Estimated.
5	*k*_1_	Total number of contacts, (effective), per day of an exposed individual.	Policy dependent
6	*b*_1_	Probability of infection between carrier(E) and susceptible individual	Estimated
7	*γ* − 1	Mean time for an individual to go from E to I.	To be fitted
8	*δ* − 1	Mean time for an individual to go from I to H compartment.	To be fitted
9	*κ*(*t*)	Mortality rate (time-dependent).	To be fitted
10	*λ*(*t*)	Recovery rate (time-dependent).	To be fitted

**Table 4:** Fitted parameters of the proposed SEIHRD model before and after 24 March.

S.No.	Coefficient	Value before 24 March	Value after 24 March
1	*β*	0.455	0.41755
2	*k*	10	5
3	*b*	0.0455	0.08351
4	*β*_1_	0.91	0.8351
5	*k*_1_	20	10
6	*b*_1_	0.0455	0.08351
7	*γ* − 1	5 days	5 days
8	*δ* − 1	4 days	7 days
9	*κ*(*t*)	0.05%	1.02%
10	*λ*(*t*)	5%	26%

## Data Availability

The data presented in this study are available on request from the corresponding author.

## References

[1] Yunfeng S., Haiwei L., Ren Z. (2021). Effects of Pandemic Outbreak on Economies: Evidence from Business History Context. Front. Public Health.

[2] The Editors of Encyclopaedia Britannica (2022). Black Death. Encyclopedia Britannica.

[3] Vaibhav B., Poonia R.C., Nagar P., Kumar S., Singh V., Raja L., Dass P. (2021). Descriptive analysis of COVID-19 patients in the context of India. J. Interdiscip. Math..

[4] Sunil C., Mittal M., Chawla M., Goyal L. (2020). Corona virus-SARS-CoV-2: An insight to another way of natural disaster. EAI Endorsed Trans. Pervasive Health Technol..

[5] Platt C. (2014). King Death: The Black Death and its Aftermath in Late-Medieval England.

[6] DeWitte S.N. (2014). Mortality risk and survival in the aftermath of the medieval Black Death. PLoS ONE.

[7] Yang W., Petkova E., Shaman J. (2013). The 1918 influenza pandemic in New York City: Age-specific timing, mortality, and transmission dynamics. Influenza and Other Respiratory Viruses. Natl. Inst. Health.

[8] Simon D., McDonald S., Roberts J. (2001). AIDS and economic growth in Africa: A panel data analysis. J. Int. Dev. J. Dev. Stud. Assoc..

[9] Frieden N.M. (1977). The Russian Cholera Epidemic, 1892–1893, and Medical Professionalization. J. Soc. Hist..

[10] Warren J. (2001). Jared Diamond, Guns, Germs and Steel: The Fates of Human Societies. J. Mil. Strateg. Stud..

[11] Keogh-Brown M.R., Smith R.D. (2008). The economic impact of SARS: How does the reality match the predictions?. Health Policy.

[12] Achonu C., Laporte A., Gardam M.A. (2005). The financialimpact of controlling a respiratory virus outbreak in a teaching hospital. Can. J. Public Health.

[13] Michele T., Bajardi P., Poletto C., Ramasco J.J., Balcan D., Gonçalves B., Perra N., Colizza V., Vespignani A. (2012). Real-time numerical forecast of global epidemic spreading: Case study of 2009 A/H1N1pdm. BMC Med..

[14] Seema J., Kamimoto L., Bramley A.M., Schmitz A.M., Benoit S.R., Louie J., Sugerman D.E., Druckenmiller J.K., Ritger K.A., Chugh R. (2009). Hospitalized patients with 2009 H1N1 influenza in the United States, April–June 2009. N. Engl. J. Med..

[15] Sharma A., Agarwal B. (2021). A cyber-physical system approach for model based predictive control and modeling of COVID-19 in India. J. Interdiscip. Math..

[16] Nicola M., Alsafi Z., Sohrabi C., Kerwan A., Al-Jabir A., Iosifidis C., Agha M., Agha R. (2020). The socio-economic implications of the coronavirus pandemic (COVID-19): A review. Int. J. Surg..

[17] The Economic Times Mirror Now. Time to Re-Open Delhi, People Will Have to Be Ready to Live with Coronavirus: C M kejri-wal. https://www.business-standard.com/article/current-affairs/time-to-reopen-delhi-live-with-virus-120050400054_1.html.

[18] Bernoulli D., Haberman S., Sibbett T.A. (1995). Essai d’une nouvelle analyse de la mortalité causée par la petite verole et des avantages de l’inoculation pour la prevenir. *Mem. Math. Phys. Acad. Roy. Sci.*
**1766**, 1–45. Histoire de l’Academie Royale des Sciences.

[19] Ogilvy K.W., McKendrick A.G. (1927). A contribution to the mathematical theory of epidemics. Proc. R. Soc. Lond. A.

[20] Dietz K., Heesterbeek J.A.P. (2002). Daniel Bernoulli’s epidemiological model revisited. Math. Biosci..

[21] Iftimie S., López-Azcona A.F., Vallverdú I., Hernández-Flix S., de Febrer G., Parra S., Hernández-Aguilera A., Riu F., Joven J., Andreychuk N. (2021). First and second waves of coronavirus disease-19: A comparative study in hospitalized patients in Reus, Spain. PLoS ONE.

[22] Lu J. (2020). A new, simple projection model for COVID-19 pandemic. medRxiv.

[23] Pierre M., Webb G. (2020). Predicting the number of reported and unreported cases for the COVID-19 epidemic in South Korea, Italy, France and Germany. https://ssrn.com/abstract=3557360.

[24] Bekiros S., Kouloumpou D. (2020). Sbdiem: A new mathematical model of infectious disease dynamics. Chaos Solitons Fractals.

[25] Victor A. (2020). Mathematical predictions for COVID-19 as a global pandemic. medRxiv.

[26] Toda A.A. (2020). Susceptible-infected-recovered (SIR) dynamics of Covid-19 and economic impact. arXiv.

[27] Caccavo D. (2020). Chinese and italian covid-19 outbreaks can be correctly described by a modified sird model. medRxiv.

[28] Jia W., Ke H., Yang S., Wenzhe C., Shengshu W., Shanshan Y., Jianwei W., Kou F., Tai P., Li J. (2020). Extended SIR prediction of the epidemics trend of COVID-19 in Italy and compared with Hunan, China. Front. Med..

[29] Chandra P.R., Dass P., Raja L., Bhatnagar V., Prasad J. (2022). The Review of Prediction Models for COVID-19 Outbreak in Indian Scenario. Proceedings of the Third International Conference on Sustainable Computing.

[30] Sameni R. (2020). Mathematical modeling of epidemic diseases; a case study of the COVID-19 coronavirus. arXiv.

[31] Kaustuv C., Chatterjee K., Kumar A., Shankar S. (2020). Healthcare impact of COVID-19 epidemic in India: A stochastic mathematical model. Med. J. Armed Forces India.

[32] Dowd J.B., Andriano L., Brazel D.M., Ro-tondi V., Block P., Ding X., Liu Y., Mills M.C. (2020). Demo-graphic science aids in understanding the spread and fatality rates of covid-19. Proc. Natl. Acad. Sci. USA.

[33] He X., Lau E.H.Y., Wu P., Deng X., Wang J., Hao X., Lau Y.C., Wong J.Y., Guan Y., Tan X. (2020). Temporal dynamics in viral shedding and transmissibility of COVID-19. Nat. Med..

[34] Amitava B., Pasea L., Harris S., Gonzalez-Izquierdo A., Torralbo A., Shallcross L., Noursadeghi M., Pillay D., Sebire N., Holmes C. (2020). Estimating excess 1-year mortality associated with the COVID-19 pandemic according to underlying conditions and age: A population-based cohort study. Lancet.

[35] Kucharski A.J., Russell T.W., Diamond C., Liu Y., Edmunds J., Funk S., Eggo R.M., Sun F., Jit M., Munday J.D. (2020). Early dynamics of transmission and control of COVID-19: A mathematical modelling study. Lancet Infect. Dis..

[36] Gaurav P., Chaudhary P., Gupta R., Pal S. (2020). SEIR and Regression Model based COVID-19 outbreak predictions in India. arXiv.

[37] Youssoufa M., Halidou A., Kapen P.T. (2020). A review of mathematical modeling, artificial intelligence and datasets used in the study, prediction and management of COVID-19. Appl. Intell..

[38] Luo J. (2020). When Will COVID-19 End? Data-Driven Prediction. Singapore University of Technology and Design. http://www.sutd.edu.sg.

[39] Liangrong P., Yang W., Zhang D., Zhuge C., Hong L. (2020). Epidemic analysis of COVID-19 in China by dynamical modeling. arXiv.

[40] Zifeng Y., Zeng Z., Wang K., Wong S., Liang W., Zanin M., Liu P., Cao X., Gao Z., Mai Z. (2020). Modified SEIR and AI prediction of the epidemics trend of COVID-19 in China under public health interventions. J. Thorac. Dis..

[41] Jinming C., Jiang X., Zhao B. (2020). Mathematical modeling and epidemic prediction of COVID-19 and its significance to epidemic prevention and control measures. J. Biomed. Res. Innov..

[42] Otunuga O.M., Ogunsolu M.O. (2020). Qualitative anal-ysis of a stochastic seitr epidemic model with multiple stages of infectionand treatment. Infect. Dis. Model..

[43] Soniya L., Sahni G., Mewara B., Kumar R. (2020). Predicting optimal lockdown period with parametric approach using three-phase maturation SIRD model for COVID-19 pandemic. Chaos Solitons Fractals.

[44] Ghosh A., Roy S., Mondal H., Biswas S., Bose R. (2022). Mathematical modelling for decision making of lockdown during COVID-19. Appl. Intell..

[45] Swapnarekha H., Behera H.S., Nayak J., Naik B. (2020). Role of intelligent computing in COVID-19 prognosis: A state-of-the-art review. Chaos Solitons Fractals.

[46] Kotwal A., Yadav A.K., Yadav J., Kotwal J., Khune S. (2020). Predictive models of COVID-19 in India: A rapid review. Med. J. Armed Forces India.

[47] Park M., Cook A.R., Lim J.T., Sun Y., Dickens B.L. (2020). A systematic review of COVID-19 epidemiology based on current evidence. J. Clin. Med..

[48] Harjule P., Rahman A., Agarwal B. (2021). A cross-sectional study of anxiety, stress, perception and mental health towards online learning of school children in India during COVID-19. J. Interdiscip. Math..

[49] Kermack W.O., McKendrick A.G. (1991). Contributions to the mathematical theory of epidemics—I. 1927. Bull. Math. Biol..

[50] National Portal of India. https://main.icmr.nic.in/content/covid-19.

[51] Covidindia Data. https://www.covid19india.org.

[52] He D., Dushoff J., Day T., Ma J., Earn D.J.D. (2013). Inferring the causes of the three waves of the 1918 influenza pandemic in England and Wales. Proc. R. Soc. B Biol. Sci..

